# FEM: feature-enhanced map

**DOI:** 10.1107/S1399004714028132

**Published:** 2015-02-26

**Authors:** Pavel V. Afonine, Nigel W. Moriarty, Marat Mustyakimov, Oleg V. Sobolev, Thomas C. Terwilliger, Dusan Turk, Alexandre Urzhumtsev, Paul D. Adams

**Affiliations:** a Physical Biosciences Division, Lawrence Berkeley National Laboratory, One Cyclotron Road, MS64R0121, Berkeley, CA 94720, USA; b Biology and Soft Matter Division, Oak Ridge National Laboratory, PO Box 2008, Oak Ridge, TN 37831, USA; c Los Alamos National Laboratory, Mail Stop M888, Los Alamos, NM 87545, USA; d Biochemistry and Molecular and Structural Biology, Jozef Stefan Institute, Jamova 39, SI-1000 Ljubljana, Slovenia; e Centre of Excellence for Integrated Approaches in Chemistry and Biology of Proteins, Jozef Stefan Institute, Jamova 39, SI-1000 Ljubljana, Slovenia; f Centre for Integrative Biology, IGBMC, CNRS–INSERM–UdS, 1 Rue Laurent Fries, BP 10142, 67404 Illkirch, France; g Département de Physique, Faculté des Sciences et des Technologies, Université de Lorraine, 54506 Vandoeuvre-lès-Nancy, France; h Department of Bioengineering, University of California Berkeley, Berkeley, CA 94720, USA

**Keywords:** Fourier map, map sharpening, map kurtosis, model bias, map improvement, density modification, *PHENIX*, *cctbx*, FEM, feature-enhanced map, OMIT

## Abstract

A method is presented that modifies a 2*m* **F**_obs_ − *D* **F**_model_ σ_A_-weighted map such that the resulting map can strengthen a weak signal, if present, and can reduce model bias and noise. The method consists of first randomizing the starting map and filling in missing reflections using multiple methods. This is followed by restricting the map to regions with convincing density and the application of sharpening. The final map is then created by combining a series of histogram-equalized intermediate maps. In the test cases shown, the maps produced in this way are found to have increased interpretability and decreased model bias compared with the starting 2*m* **F**_obs_ − *D* **F**_model_ σ_A_-weighted map.

## Abstract 2

The non-iterative feature-enhancing approach improves crystallographic maps’ interpretability by reducing model bias and noise and strengthening the existing signal.

## 1. Introduction   

An electron (or nuclear) density map is typically an important step in obtaining an atomic representation of a crystal structure, or the map itself may serve as the model of the contents of the crystal. In either case the quality of the map affects its utility. In this work, we consider a desirable map to be one that accurately represents the actual electron (or nuclear) density in an average unit cell of the crystal. There are at least three different factors that affect the quality of crystallographic maps and their interpretation.

(i) *Errors in and incompleteness of the data*. The finite resolution and the incompleteness of measured reflections and errors in experimental data and crystallographic model parameters are major contributors to poor map quality. These errors may obscure or corrupt the signal, making meaningful interpretation difficult or even impossible.
(ii) *Signal weakness*. Another source of difficulty in map interpretation is that not all of the signal presented in the map has the same strength. For example, a strong signal (a high peak of electron density in the map) arising from a heavy-atom derivative may easily obscure a very weak signal (a low electron density) arising from a partially occupied mobile ligand, an alternative conformation of a residue side chain or even H atoms. It is common that such weak signal may be near or below the perceived noise level in a map.
(iii) *Model bias*. Crystallographic maps are often calculated using model phases or a combination of model phases with some experimentally obtained phase information. Errors in the atomic, or non-atomic (bulk solvent, for instance), model can result in noise features in the map that appear very similar to atomic features and in turn may be erroneously interpreted as such.

In practice, all three types of map imperfections may be present together. They make the interpretation of crystallo­graphic maps ambiguous, nontrivial or non-unique at typical macromolecular resolutions of approximately 1–4 Å.

A large amount of methodology has been developed to improve crystallographic maps. These methodologies can be roughly divided into four categories.

(i) Those focusing on the choice of various weighting schemes (Luzzati, 1953 ▶; Woolfson, 1956 ▶; Sim, 1959 ▶; Raman, 1959 ▶; Ramachandran & Raman, 1959 ▶; Srinivasan, 1961 ▶; Ramachandran & Srinivasan, 1961 ▶, 1970 ▶; Main, 1979 ▶; Vijayan, 1980 ▶; Urzhumtsev *et al.*, 1996 ▶; Vellieux & Dijkstra, 1997 ▶; Read, 1997 ▶), with the most broadly used approach being σ_A_-weighted syntheses (Read, 1986 ▶).
(ii) Obtaining maps derived from ensembles of perturbed models or structure factors (Agarwal & Isaacs, 1977 ▶; Lunin & Urzhumtsev, 1984 ▶; Lunin *et al.*, 1985 ▶; Baker *et al.*, 1993 ▶; Perrakis *et al.*, 1997 ▶; Rice *et al.*, 1998 ▶; Reddy *et al.*, 2003 ▶; Pražnikar *et al.*, 2009 ▶; Lang *et al.*, 2014 ▶).
(iii) Various kinds of OMIT maps (Bhat & Cohen, 1984 ▶; Bhat, 1988 ▶; Hodel *et al.*, 1992 ▶; Gunčar *et al.*, 2000 ▶; Vellieux & Dijkstra, 1997 ▶; Terwilliger *et al.*, 2008 ▶; Pražnikar *et al.*, 2009 ▶; Cowtan, 2012 ▶).
(iv) Density-modification techniques (for reviews, see Podjarny *et al.*, 1996 ▶; Zhang *et al.*, 2006 ▶; Cowtan, 2010 ▶).

All of these methods aim to address one or a few specific issues pertinent to map quality, but not all or at once. Some of them are quick to compute (*e.g.* σ_A_ or kick maps), while the calculation of others (*e.g.* OMIT, SA OMIT or iterative-build OMIT maps) is more computationally intensive and includes more complex calculations such as refinement and/or model building that require problem-specific parameterization. Some may rely on information that needs to be either provided by a user or estimated, such as values of the *F*_000_ reflection for RAPID/END or maximum-entropy maps or solvent content for density modification. OMIT maps attempt to address one problem (model bias) while potentially introducing other perturbations into the map. Indeed, the calculation of an OMIT map involves removing parts of the model, so one may expect that the quality of the resulting map might be worse than that of the original map (although the model bias may be reduced). Density-modification methods are generally tailored to the beginning of structure determination and are good at bulk map improvements and eliminating gross errors, while these methods may be harsh on weak local signal (such as densities arising from partially occupied mobile ligands).

This manuscript describes a procedure that improves a 2*m* **F**_obs_ − *D* **F**_model_ σ_A_-weighted map (Read, 1986 ▶). The result is a new map that possesses a reduced level of noise and model bias and that also shows enhancement of weak features, often bringing them onto the same scale as the strong features. We call this map a feature-enhanced map (FEM). The new map is expected to contain less noise and have a more easily interpretable available signal both for human interpretation and for model-building software. While in this work we focus on 2*m* **F**_obs_ − *D* **F**_model_ maps, the procedure can be extended to other kinds of maps. The only inputs to the current procedure are measured data (*I*_obs_ or *F*_obs_), measurement uncertainties [σ(*I*_obs_) or σ(*F*_obs_)] and the current atomic model. Optionally, additional phase information can be used in the form of Hendrickson–Lattman coefficients. The procedure does not involve sophisticated machinery such as model building or refinement. Specifically, the novel, new or redesigned methods implemented as part of the FEM calculation procedure include the following.

(i) A protocol for the calculation of a composite residual OMIT map.
(ii) Using map kurtosis as a measure of crystallographic map sharpness and as an optimization goal for *B*-factor sharpening.
(iii) The application of the histogram-equalization method to crystallographic maps for the purpose of improvement of map interpretation.
(iv) The application of the unsharp masking technique to crystallographic maps.
(v) Efficient map randomization, computer memory-efficient storage of many maps simultaneously and map combination.
(vi) A procedure for filling missing reflections.
(vii) A method of low-volume density elimination based on connectivity analysis and multiple-cutoff map contouring.

## 2. Methods   

Conceptually, the FEM method consists of three key stages. Firstly, the starting map is defined and calculated. In the second step, the starting map is randomized in a variety of different ways; these include randomizing the initially calculated map itself and varying the way in which the map is calculated. This procedure generates an ensemble of slightly different maps that are then combined into one single map with the goal of reducing noise and model bias. As expected, combining maps tends to blur density peaks and therefore map sharpening of some form is helpful. The final step involves map modification to equalize the strength of the signal throughout the entire unit-cell volume, so the weak features become roughly the same magnitude as the strong features. This step is not selective and could enhance both the signal and noise; therefore, it is essential to eliminate the noise as much as possible in the first two steps.

**Figure 1:**
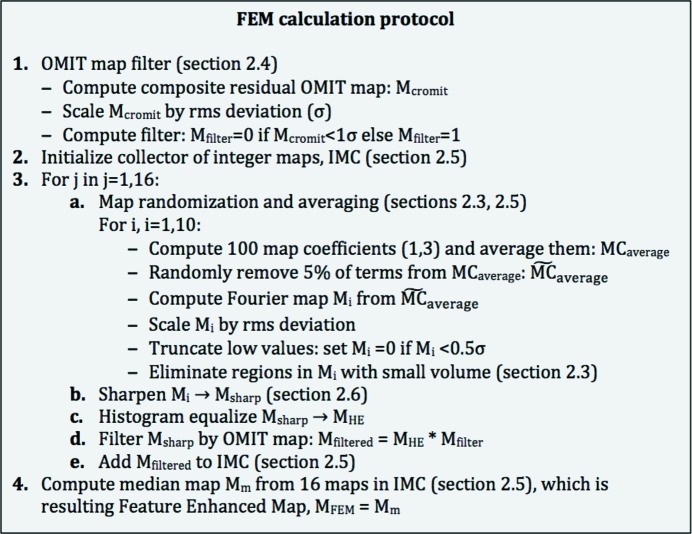
FEM protocol. The individual steps are explained in the corresponding sections of the manuscript.

The implementation of this concept is detailed in Fig. 1 ▶, with the individual steps explained in the sections below. The protocol is empirical and is the result of experimentation with a wide range of procedures.

### 2.1. Calculation of a map   

The map that is subjected to the FEM procedure is computed with the following complex Fourier coefficients (given as an amplitude and a phase),
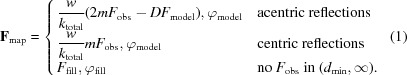
The total model structure factors, **F**_model_ = *k*_total_(**F**_calc_ + *k*_mask_ **F**_mask_), are calculated as described in Afonine, Grosse-Kunstleve *et al.* (2013 ▶). Prior to **F**_model_ calculation, the maximal possible common isotropic *B* factor, *B*_max_, is subtracted from all atoms (*B*_max_ is defined such that after subtraction all atoms still have non-negative definite *B* factors; see §§[Sec sec2.6.1]2.6.1 and [Sec sec3]3 for further details). After overall scaling has been performed, *B*_max_ becomes part of an overall anisotropic scale factor *k*_total_ (see Afonine, Grosse-Kunstleve *et al.*, 2013 ▶ for definitions). Dividing the map coefficients **F**_map_ by *k*_total_ sharpens the map and removes anisotropy. Unit weights *w* are used by default. For acentric and centric reflections (the first two rows in equation 1), σ_A_-weighted (Read, 1986 ▶) anisotropy-removed and sharpened Fourier map coefficients corresponding to measured *F*_obs_ are calculated. The (*F*_fill_, ϕ_fill_) terms correspond to unmeasured (missing) reflections, while ϕ_model_ are either model phases (phases of **F**_model_) or model phases combined with experimental phase information. The weights *m* and *D* are calculated as described in Read (1986 ▶) and Urzhumtsev *et al.* (1996 ▶).

A residual (difference) synthesis is computed similarly to (1), 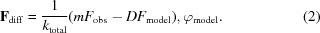



### 2.2. Modeling missing reflections   

Let *d*_min_ be the highest resolution limit of a set of *F*_obs_. It has been demonstrated that if the terms corresponding to unmeasured reflections *F*_obs_ in the resolution range (*d*_min_, ∞) are missing from the map calculation then the map quality is degraded (Lunin, 1988 ▶; Urzhumtsev *et al.*, 1989 ▶; Lunin & Skovoroda, 1991 ▶; Tronrud, 1996 ▶; Cowtan, 1996 ▶; Lunina *et al.*, 2002 ▶; Urzhumtseva & Urzhumtsev, 2011 ▶). To mitigate this negative effect, terms corresponding to missing *F*_obs_ are replaced (often referred to as ‘filled in’) with values (*F*_fill_, ϕ_fill_) (Murshudov *et al.*, 1997 ▶; Lunina *et al.*, 2002 ▶; Altomare *et al.*, 2008 ▶; Sheldrick, 2008 ▶). Ignoring missing reflections is essentially equivalent to postulating that their values are all zero (**F**_fill_ = 0). We have implemented two ways of approximating the terms corresponding to the missing *F*_obs_.

The first method uses the density-modification functionality of *RESOLVE* (Terwilliger, 2003 ▶). A feature of density modification is that it can be used to estimate the amplitudes as well as the phases of reflections that are missing. In the statistical density-modification approaches implemented in *RESOLVE*, these amplitudes and phases are those that maximize the likelihood (plausibility) of the map based on such features as the flatness of the solvent, noncrystallographic symmetry and density distributions (Terwilliger, 2003 ▶). The use of density modification to estimate missing amplitudes consists of carrying out several cycles of density modification and finally using the map-based amplitudes for the missing reflections in the resolution range (*d*_min_, ∞).

The second method for the estimation of missing amplitudes uses the available atomic model. A key element of this second approach is that atomic model truncation is carried out to eliminate unreliably placed atoms so that they do not feed back into the map through **F**_fill_. Firstly, a Fourier map (1) is calculated with **F**_fill_ all zero and normalized based on the r.m.s. deviation of the density in the map. Each atom is then scored against this map and the average density at the atomic centers (ρ_ave_) is noted. Atoms that have a map correlation worse than 0.7 or have a density value at the atom center of less than min{½ρ_ave_, 1} are removed. Since we consider a situation close to the final structure, only a relatively small part of the atoms may be filtered out. This truncated model is then used to calculate **F**_model_ in the resolution range (*d*_min_, ∞). A bulk-solvent contribution to **F**_model_ is calculated as described in Afonine *et al.* (2005 ▶) and the mask is calculated from the truncated atomic model. This allows optimal *k*_sol_ and *B*_sol_ values to be obtained, as well as the overall anisotropic scale factor. The **F**_model_ values for the full resolution range are calculated and the missing reflections that are part of **F**_model_ are then assigned to **F**_fill_. We note that using typical values of *k*_sol_ and *B*_sol_ of 0.35 e Å^−3^ and 46 Å^2^, respectively (Fokine & Urzhumtsev, 2002 ▶), is sub­optimal, occasionally resulting in maps with pronounced artifacts in the bulk-solvent region.

**Figure 2:**
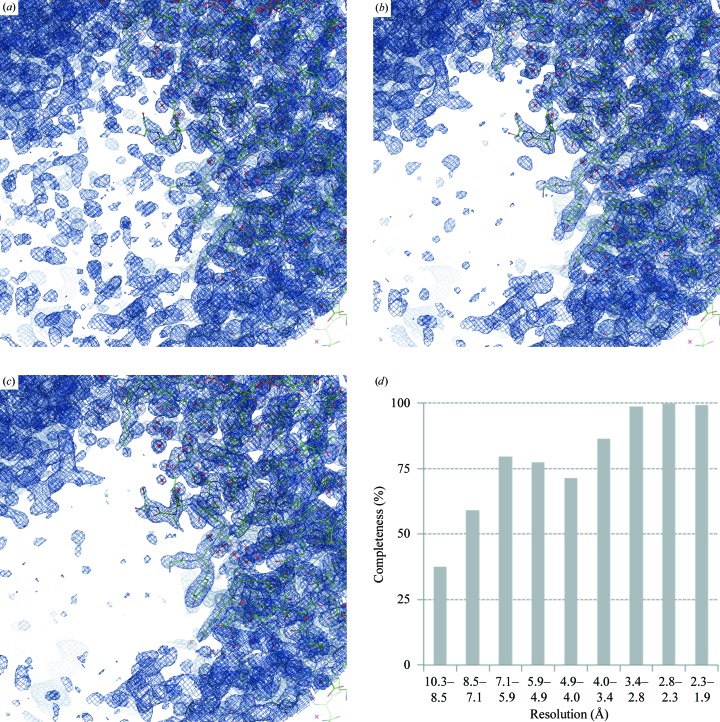
The effect of missing data and its restoration for PDB entry 1nh2: maps calculated using (1) with (*a*) **F**_fill_ = 0 (1.0σ), (*b*) **F**_fill_ derived from a *RESOLVE* density-modified map (1.1σ) and (*c*) **F**_fill_ derived from the model as described in §[Sec sec2.2]2.2 (1.0σ). (*d*) Resolution-bin completeness of the diffraction data for PDB entry 1nh2. Note the very poor completeness at low resolution and the rather good overall completeness, which is 95% in the range (1.9, ∞). Log-scale binning is used as described in Afonine, Grosse-Kunstleve *et al.* (2013 ▶), which efficiently highlights poor low-resolution completeness. See §[Sec sec2.9]2.9 regarding the choice of map-contouring levels.

We find that estimating values for missing reflections in this way generally reduces map noise. As an example, Fig. 2 ▶ shows the map calculated with (1) using **F**_fill_ = 0 as well as maps where missing terms (**F**_fill_) were derived either using statistical density modification or based on a working model. PDB (Bernstein *et al.*, 1977 ▶; Berman *et al.*, 2000 ▶) entry 1nh2 was used in this example.

### 2.3. Removal of noise and model bias by map randomization and combination   

The rationale behind map randomization followed by combination of the resulting perturbed maps is based on the hypothesis that map artifacts and noise are more affected by randomization than the signal in the map (Kirillova, 2008 ▶). In this way, the artifacts and noise might be reduced compared with the signal by map averaging or some other statistical treatment of the resulting maps. This approach is also the basis of kick maps (Gunčar *et al.*, 2000 ▶; Turk, 2013 ▶).

**Figure 3:**
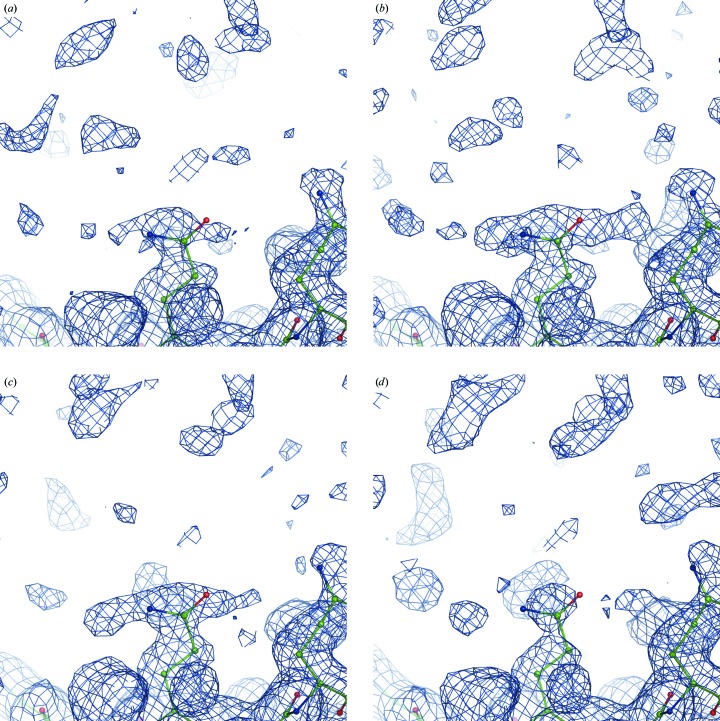
Fourier maps calculated with (1): (*a*) *w* = 1 (0.5σ), (*b*, *c*) *w* computed using (3) (0.45σ), (*d*) the same as (*c*) but with 5% of the terms omitted. Note that the signal (the density around the atoms) is similar while the noise (the density further away from the atoms) is reshuffled in all four cases. See §[Sec sec2.9]2.9 regarding the choice of contouring threshold levels.

Here, we took a different route to achieve a similar goal. Empirically, we found that the use of a weighting scheme in (1) such as
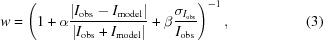
possesses the desired property of perturbing the map noise more than the signal. Here, α and β are constants individually defined for each reflection, *I*_obs_ and *I*_model_ are the observed and calculated structure-factor intensities, respectively, and 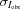 are the experimental uncertainties of the measured *I*_obs_. The sum spans over all reflections. If α and β are picked randomly for each reflection, then each new set of Fourier map coefficients (1) will have a unique set of weights *w* and therefore will generate a unique Fourier map. We found that a useful range of α and β values is between 0 and 5, allowing the generation of maps that are sufficiently different in noise but that have the signal perturbed only a little, as illustrated in Figs. 3 ▶(*a*), 3 ▶(*b*) and 3 ▶(*c*).

**Figure 4:**
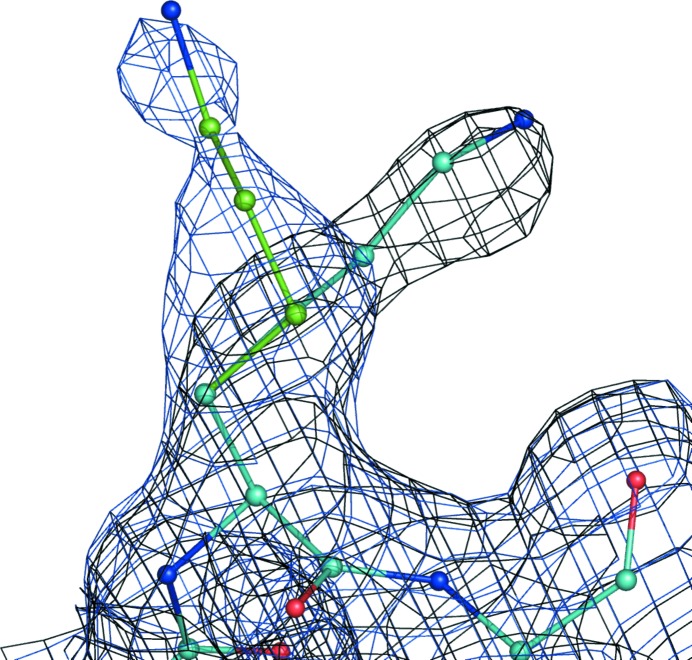
The correct residue Lys107 (chain *L*) of PDB entry 1f8t (the rotamer on the left) is shown as a blue map calculated using (1) with *w* = 1. The same residue side chain switched to an incorrect rotamer (right) is shown as a black map calculated using (1) with weights from (3) with δ = 1.5. Both maps are contoured at 1σ. Clearly, the black map is model-biased as it follows the wrong side-chain orientation and otherwise coincides with the correct (blue) map.

The idea of using the weights in (3) was inspired by the empirical weighting scheme used in *SHELX* (Sheldrick, 2008 ▶),
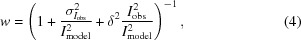
where the parameter δ is a small numerical value. By defining δ per reflection similarly to (3) one can also perturb the map. However, we noticed that small values of δ (0 < δ < 1) do not change the maps visibly compared with using unit weights *w*, while using δ > 1 results in severely model-biased maps, as exemplified in Fig. 4 ▶.

In our approach, an ensemble of Fourier map coefficients is generated by repeated (*N* times) calculation of individual sets of map coefficients using equations (1) and (3) with **F**_fill_ obtained as described in §[Sec sec2.2]2.2.

Since an ensemble of Fourier maps (1) is generated, this provides an opportunity to randomize the generation of **F**_fill_ each time the new map is calculated. This is achieved by randomly removing an additional 10% of the atoms and randomly drawing *k*_sol_ and *B*_sol_ values from the distributions (0, 0.01, …, 0.4) and (20, 25, …, 80), respectively, for the purpose of **F**_fill_ calculation (§[Sec sec2.2]2.2).

A combined Fourier map is then obtained in the following way (step 3a in Fig. 1 ▶). Firstly, *N* calculated sets of Fourier map coefficients are split into *n* groups each containing *N*/*n* sets of map coefficients. The map coefficients in each group are then averaged, 5% of the map coefficients (reflections) are randomly removed from each averaged set of coefficients and a Fourier map is calculated with the remaining reflections. This is repeated for each group of map coefficients, resulting in *n* Fourier maps (each with 5% of the reflections randomly removed) that are then averaged in real space to yield a single map. Fig. 3 ▶ illustrates the use of the randomized weights (3) in the calculation of a map using (1).

**Figure 5:**
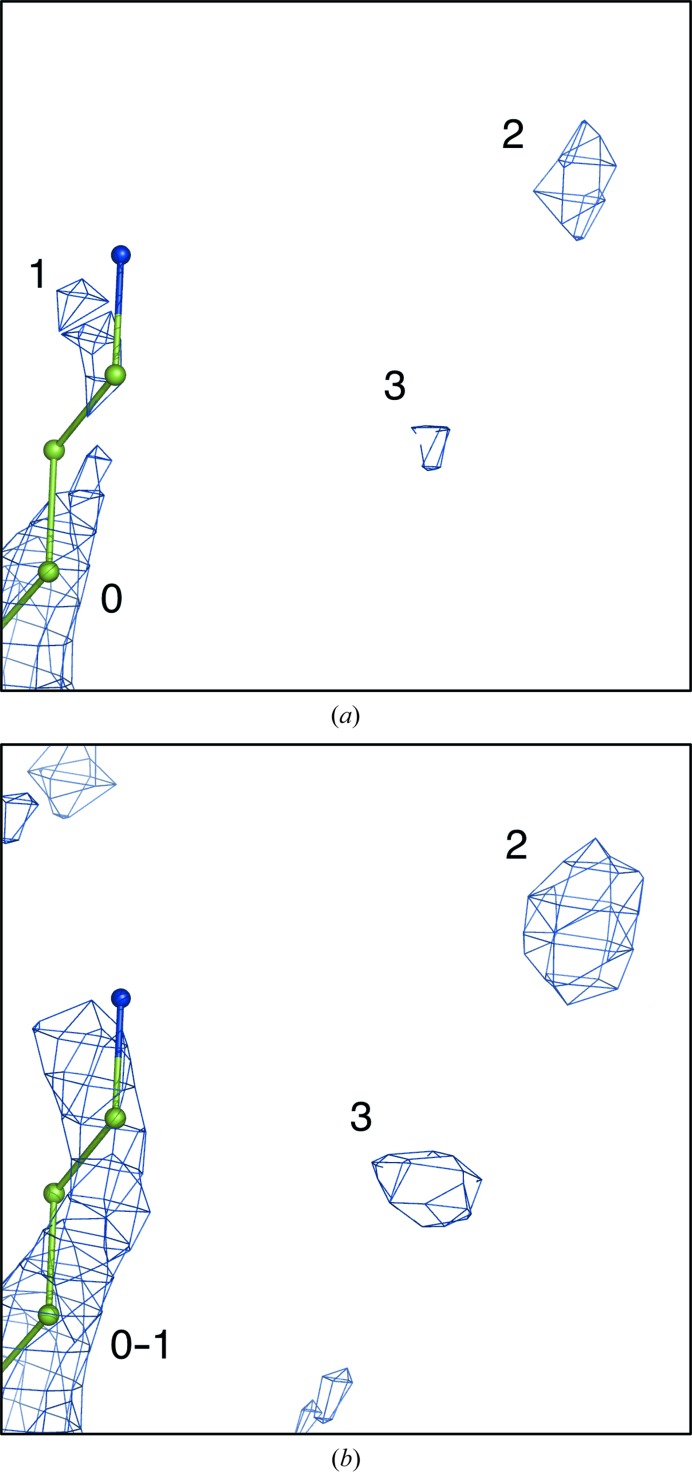
Schematic illustration of the elimination of noise peaks. (*a*) Map contoured at a threshold level *t*_1_: blobs 1, 2 and 3 are selected for elimination, while blob 0 is retained. (*b*) Map contoured at a threshold level *t*_2_ = *t*_1_ − δ. We note that blob 1 merges with blob 0 and is therefore retained, while the larger blobs 2 and 3 are removed.

The map-averaging procedure as described above reduces or even completely eliminates a large number of the noise peaks, but not all. Some persistent noise may remain, and some very low map values (for example, peaks smaller than ρ_cut_ = 0.5σ) will also always be present in the averaged map. Since later steps of the FEM procedure include nonselective equalization of map values, where the noise may be enhanced as well as the signal, it is essential that these undesirable map values are largely eliminated. This is achieved by the following two steps. One step truncates low map values at a chosen cutoff. The other identifies connected regions of the electron-density map (referred to as blobs) that have a smaller volume than a typical volume of a reliably placed atom at some threshold level *t*_1_ that can be thought of as noise and considered for elimination. Moreover, we would not only like to eliminate those blobs shown at a threshold *t*_1_, but also we want to remove their ‘roots’: these same blobs as they appear at a lower threshold *t*_2_ = *t*_1_ − δ. Lowering the threshold to *t*_2_ may result in some blobs that were originally identified for elimination merging with blobs that were not considered for elimination. Such blobs are retained. Schematically, this is illustrated in Fig. 5 ▶. Fig. 5 ▶(*a*) shows blobs numbered 0, 1, 2, 3 at a threshold level *t*_1_, where blobs 1, 2 and 3 are selected for elimination based on their volume. Fig. 5 ▶(*b*) is the same as Fig. 5 ▶(*a*) but contoured at a threshold *t*_2_. Here, we will remove blobs 2 and 3, but blob 1 will be kept since it merges with blob 0 that was not selected for elimination. The map-connectivity analysis algorithm used for the identification of blobs has been described by Lunina *et al.* (2003 ▶).

### 2.4. Composite residual (difference) OMIT map   

An important element in the FEM procedure is restricting the final map to regions where there is convincing evidence of density. This is achieved by using a composite OMIT map. In contrast to the classical approach for calculating a composite OMIT map (Bhat & Cohen, 1984 ▶; Bhat, 1988 ▶), we implemented a procedure that is similar to that described by Cowtan (2012 ▶). The main differences from the existing approaches are that instead of omitting actual atoms in each omit box we zero the **F**_model_ synthesis in each box (omitting both the atomic and bulk-solvent contributions) and compute modified 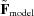 structure factors from it that are in turn used to restore the density in the box by calculating a residual synthesis (2). All maps are computed and manipulated in the asymmetric unit (Grosse-Kunstleve *et al.*, 2011 ▶).

We use the OMIT map as a filter: all grid nodes in the OMIT map with values below a certain threshold are set to zero and the remainder are set to one. Intermediate maps of the FEM procedure are multiplied by the binarized OMIT map (Fig. 1 ▶). The threshold used to binarize the OMIT map is a parameter of the procedure and by default is 1σ.

**Figure 6:**
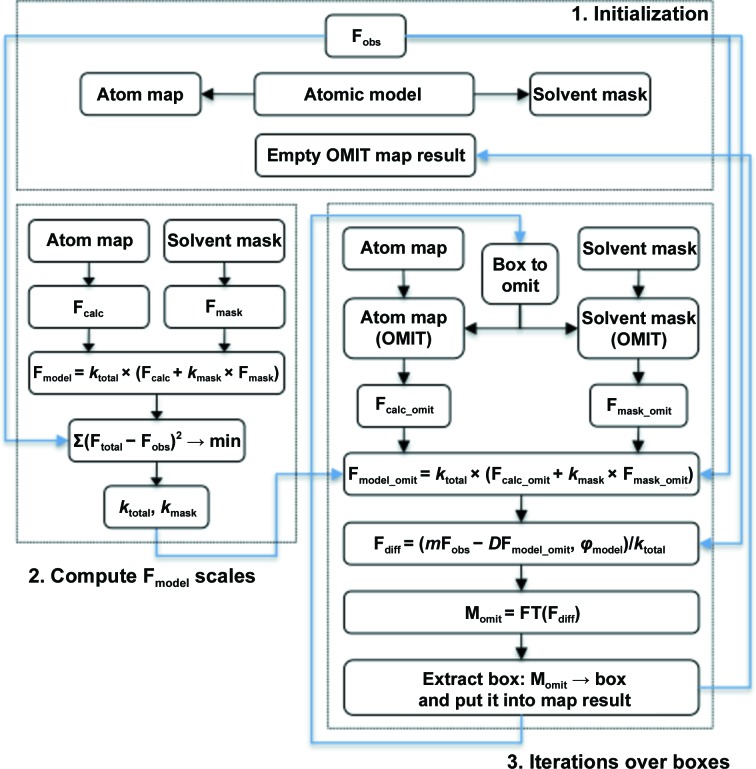
Composite residual OMIT map-calculation workflow. See §[Sec sec2.4]2.4 for details.

The procedure used to calculate the OMIT map consists of three principal stages (Fig. 6 ▶). The inputs to the procedure are the observed structure factors (*F*_obs_) and the atomic model. Firstly, a sampled model density map and bulk-solvent mask are calculated from the atomic model. At this stage an empty (initialized with zeros) map is defined. By the end of the calculations this map will be the resulting composite OMIT map. Next, the model density and bulk-solvent mask are Fourier-transformed, yielding structure factors **F**_calc_ and **F**_mask_, and scales necessary to calculate **F**_model_, *k*_total_ and *k*_mask_ are then obtained as described in Afonine, Grosse-Kunstleve *et al.* (2013 ▶). The final stage consists of a loop over boxes that cover the entire asymmetric unit. For each box, a larger box (wide box) is defined by expanding the original box by one grid node in each direction (the ‘neutral’ volume in Bhat, 1988 ▶); the corresponding volumes inside the wide box in the model map and bulk-solvent mask are then set to zero. The modified model map and bulk-solvent mask are used to calculate the corresponding structure factors **F**_calc_omit_ and **F**_mask_omit_. In turn, these structure factors and the previously calculated scales *k*_total_ and *k*_mask_ are used to calculate **F**_model_omit_, the coefficients *m* and *D* and the residual Fourier map coefficients **F**_diff_ (2), which are then Fourier-transformed into a residual synthesis. Finally, the smaller box corresponding to the wide box is extracted from this synthesis and placed into the initially pre-emptied OMIT map result. The final composite residual OMIT map is obtained upon the completion of iterations over all boxes.

**Figure 7:**
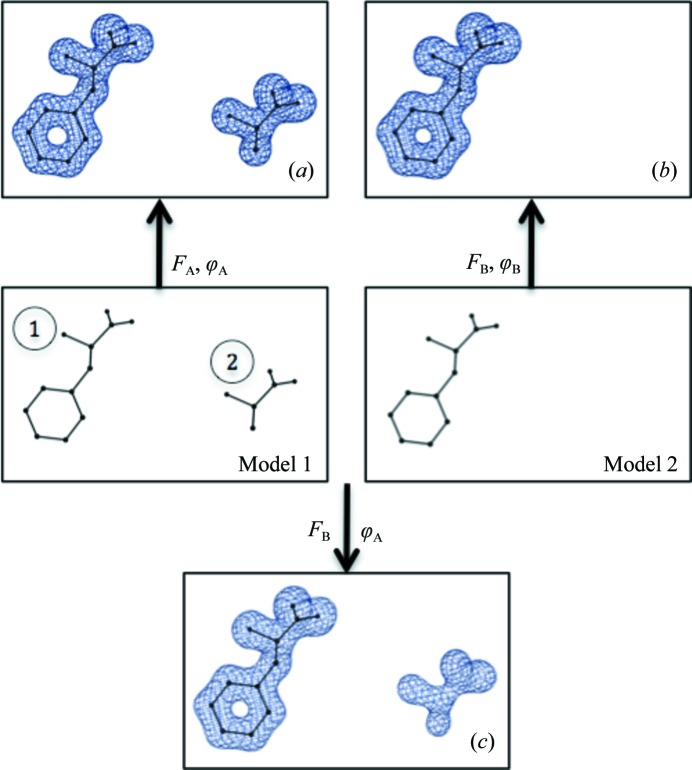
Testing the performance of the OMIT map-calculation procedure. Model 1 consists of two residues, 1 and 2, placed in a *P*1 box and is used to calculate the (*F*_A_, ϕ_A_) synthesis (*a*). Model 2 consists of one residue, 1 (otherwise identical to model 1), and is used to calculate the (*F*_B_, ϕ_B_) synthesis (*b*). Amplitudes *F*_B_ and phases ϕ_A_ are used to compute synthesis (*c*). All syntheses are contoured at 3σ. The positive map around residue 2 in (*c*) is purely model bias.

To examine the efficacy of the procedure, we performed the following numerical experiment. Firstly, we created an example that generates a model-biased map as detailed in Fig. 7 ▶. We then applied the OMIT procedure described above. The inputs to the procedure were the amplitudes of structure factors **F**_B_ and model 1 that contains both residues. The local map correlation coefficient for residue 2 was 0.99 calculated using the (F_B_, ϕ_A_) synthesis and was smaller than 0.01 for the OMIT map. This indicates that information about residue 2 is not visibly present in the OMIT map.

### 2.5. Map combination   

The goal of this step is to combine the values for a series of *N* maps obtained as variations around the initial conditions. In particular, the maps corresponding to the same structure and the same crystal are calculated on the same grid and the Fourier coefficients are different but not substantially different. Usually *N* is of the order of 8–16. The combination is performed independently for each grid node. Two problems need to be addressed: (i) how to store all *N* maps simultaneously in a memory-efficient way and (ii) what statistical procedure to use to extract a ‘signal’ from *N* map values in a given grid node (having *N* maps implies that each grid node has *N* values associated with it).

Typically, map values are stored as a set of four-byte or eight-byte real values (eight bytes in *cctbx*; Grosse-Kunstleve & Adams, 2002 ▶; Grosse-Kunstleve *et al.*, 2002 ▶), which means that having more than one or two maps simultaneously in memory may be prohibitive. To address this issue, we propose a way to convert real-value maps into one-byte integer-value maps (integer*1 in Fortran or uint8_t or unsigned char in C++). In turn, this opens the possibility of having eight integer-value maps that would occupy as much memory as one eight-byte real-value map. Assuming that two real-value maps are the maximum that could be held in memory simultaneously, we arrived at 16 as the maximum number of integer-value maps that would be used.

Firstly, we scale these maps in quantile ranks (the same procedure as used below in applying histogram equalization; see §[Sec sec2.7]2.7 for details). The resulting maps vary from 0 to 1. If the original map has been truncated to be flat (set to zero) below some threshold (σ_0_), then the new map will be flat below some *q*_0_ < 1 value. We then convert these maps into integer numbers in the range (0, 255) as follows:
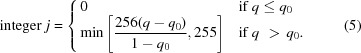
Note that multiplying by 256 instead of 255 is computationally faster.

Now for each grid node we have *N* integer values *j*_1_, *j*_2_, …, *j*_*N*_ in the range (0, 255), where *N*, as noted above, is 16. There are a number of options to analyze an array of *N* values. Since outliers may influence the average value, we decided to use the most frequent value (the mode), which is the most persistent value in a given node. Given the rather small set of data points, 16, spread across the range (0, 255), it is likely that none of the 16 numbers will coincide exactly, making it impossible to calculate the mode. To overcome this problem, working with one grid node at a time, we transform its 16-integer values into a real-value array of length 256,
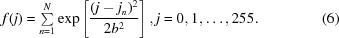
Here, each of 16 values *j*_*n*_ is blurred by a Gaussian. By trial and error, we found that values of *b* between 2 and 5 are optimal for such blurring. We then search for the argument *j*_max_ of the global maximum of the function *f*(*j*); note that *f*(*j*) is an integer-argument real-value function. Finally, we construct a quadratic interpolation for *f*(*j*_max_ − 1), *f*(*j*_max_), *f*(*j*_max_ + 1) and take the real argument *x*_max_ of the maximum of this interpolation as the final value which is assigned to this grid node in the output combined map. Obviously, the resulting map may be scaled again in quantile ranks or in r.m.s. deviations.

Two particular situations are considered separately. The first is when all values *j*_1_, *j*_2_, …, *j*_*N*_ are very different and the function *f*(*j*) has *N* peaks of equal height (equal to one or marginally higher). We consider that the synthesis does not have any information in this node and we assign it the minimal possible value, *i.e.* zero. This means that the map region composed from such points will show no structural features at any cutoff level.

Secondly, the function may have several (at least two) strong peaks of approximately equal height, *i.e.* the values *j*_1_, *j*_2_, …, *j*_*N*_ form a few groups (clusters). By default, we take the peak with the smallest argument, in other words we generate the least noisy synthesis at the risk of a possible loss or decrease of signal; we call such a synthesis ‘the minimum synthesis’. Alternatively, one can generate ‘the maximum synthesis’, where the peak with the maximum argument is taken instead. This synthesis contains the maximum signal at the risk that some noise may be highlighted.

### 2.6. Enhancing the signal by map sharpening   

Combination of several maps into one single map may result in smearing of the peaks corresponding to atoms in the structure. In addition, density may be weak owing to thermal or static disorder, as well as owing to low completeness or low resolution. For example, density corresponding to two macromolecular chains that are near to each other may merge into continuous density if the data resolution is relatively low or/and the corresponding atoms have large atomic displacement parameters. In the FEM procedure we use two methods of map sharpening to improve the map interpretability: exponential (*B*-factor) sharpening and unsharp masking.

#### 2.6.1. Global map sharpening: *B*-factor (exponential) sharpening   

*B*-factor sharpening of the map consists of multiplying the Fourier map coefficients in (1) by an exponential function of resolution exp(−*B*_sharp_|**s**|^2^/4), where **s** is a reciprocal-space vector and *B*_sharp_ is a sharpening *B* value. This technique has been shown to be useful in improving the interpretability of low-resolution maps (see Brunger *et al.*, 2009 ▶ and references therein). Obtaining an optimal *B*_sharp_ value is not straightforward because there is no mathematical criterion available that quantifies the map improvement as a function of the choice of *B*_sharp_ value. Typically, an optimal *B*_sharp_ is found by a trial-and-error method consisting of systematically sampling a range of plausible *B*_sharp_ values. The best value provides a reasonable compromise between signal improvement and increased noise, which is typically judged by eye. Other empirical approaches exist (DeLaBarre & Brunger, 2006 ▶). Here, we use the kurtosis (Abramowitz & Stegun, 1972 ▶) of the map to quantify the sharpness of the map features (‘peakedness’) and thus determine an optimal *B*_sharp_,
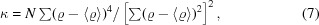
where the sums span over all map grid points, *N* is the number of grid points and 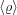 is the average density. Values of *B*_sharp_ are tested in a defined range and the value that maximizes the map kurtosis is selected. Others have also pointed out that kurtosis may be used to characterize crystallographic maps (see, for example, Lamzin & Wilson, 1993 ▶; Pai *et al.*, 2006 ▶).

**Figure 8:**
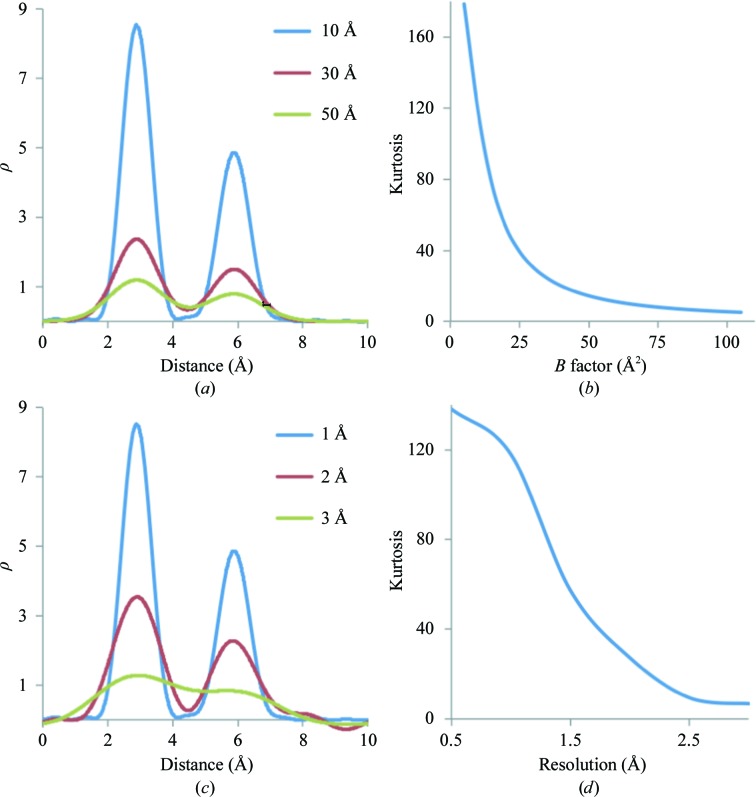
Relationships between map kurtosis and model *B* factor and data resolution. (*a*) Fourier map values of resolution 1.5 Å along the Mg—O bond shown for three selected *B*-factor values: 10, 30 and 50 Å^2^. (*b*) Map kurtosis shown as a function of the *B* factor. (*c*) Fourier map values of resolutions 1, 2 and 3 Å along the Mg—O bond. (*d*) Map kurtosis shown as a function of resolution.

Below, we provide the basis for identifying map kurtosis as a metric for selecting *B*_sharp_. We examined how data and model properties, *e.g.* resolution and *B* factors, are related to map kurtosis and why it may be a good measure to use in selecting *B*_sharp_. For this, we used a test example of two atoms, Mg and O, placed inside a 10 × 5 × 5 Å box in space group *P*1 at approximately 3 Å distance from each other. Each atom has unit occupancy. Fig. 8 ▶(*a*) shows the distribution of 1.5 Å resolution Fourier syntheses values along the Mg—O vector corresponding to three cases with all atoms having *B* factors equal to 10, 30 and 50 Å^2^, respectively. As expected, the larger the *B* factor the more smeared out the peaks are. Sampling a larger *B*-factor range, we plotted map kurtosis as function of *B* (Fig. 8 ▶ *b*). We observe that larger *B* factors smear peaks more and diminish the map kurtosis. In the next test, we varied the resolution of the synthesis, keeping the *B* factor fixed at 10 Å^2^. Fig. 8 ▶(*c*) shows Fourier syntheses values along the Mg—O vector corresponding to 1, 2 and 3 Å resolution, respectively. We also plot map kurtosis as a function of resolution (Fig. 8 ▶ *d*). We observe that lowering the resolution smears the peaks and also lowers the kurtosis of the map.

**Figure 9:**
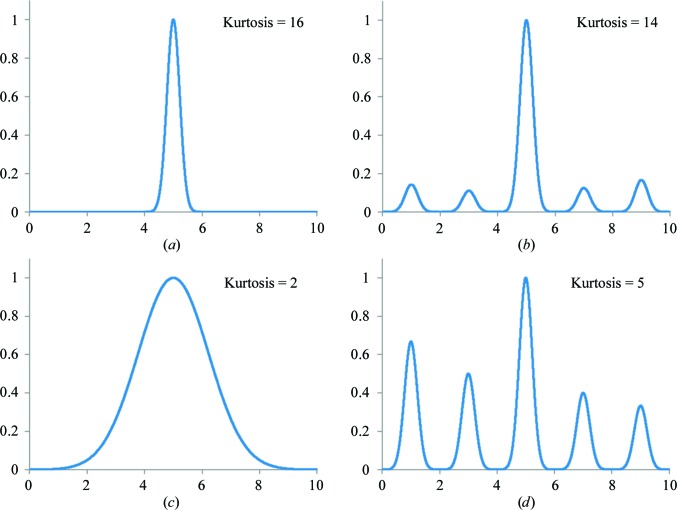
Illustrations of kurtosis for four different functions.

From the two numerical experiments presented above, we surmised that map kurtosis might be a good measure of not only the map sharpness but also of the heights of peaks in the map. Fig. 9 ▶ illustrates kurtosis using four scenarios: one sharp peak (Fig. 9 ▶ *a*), the same sharp peak and some noise (Fig. 9 ▶ *b*), a smeared peak (Fig. 9 ▶ *c*) and a sharp peak and stronger noise (Fig. 9 ▶ *d*). Clearly, broadening the peak lowers the kurtosis as well as adding noise. This example also illustrates the effect of *B*-factor sharpening and its relationship to kurtosis. Indeed, *B*-factor sharpening enhances both the signal and the noise.

**Figure 10:**
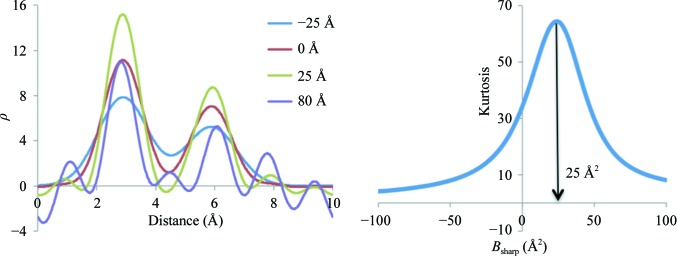
Left, distribution of Fourier map values of resolution 1.5 Å along the Mg—O bond corresponding to different sharpening *B* factors (*B*_sharp_). Right, map kurtosis as function of *B*_sharp_.

To explore this further, we performed another test using the same construct of two atoms as above. Firstly, we set the *B* factors of both atoms to 25 Å^2^ and calculated a 1.5 Å resolution synthesis. We then sampled sharpening *B* factors, *B*_sharp_, in the (−100, 100 Å^2^) range, *B*_sharp_ scaling the map coefficients for each trial as described at the beginning of this section and computing the kurtosis of the corresponding synthesis. Fig. 10 ▶(*a*) shows the result of applying four selected *B*_sharp_ values and Fig. 10 ▶(*b*) shows the dependency of map kurtosis on *B*_sharp_. We note that the sharpening *B* value that results in a maximal peak enhancement but also a minimal increase in noise is that which offsets the effective *B* factor of the two atoms to zero: a sharpening *B* value of 25 Å^2^. This value also corresponds to the peak of map kurtosis shown in Fig. 10 ▶(*b*).

From this, we conclude that map kurtosis may be a good metric for map sharpening and can be used to obtain the optimal *B*_sharp_ value. Empirically, we note that it is most effective if map noise is reduced before *B*-factor sharpening by density modification or by map combination as described above. §[Sec sec3]3 provides more examples using experimental data.

#### 2.6.2. Local map sharpening: unsharp masking   

Unsharp masking (see, for example, McHugh, 2005 ▶) is a digital image-processing technique that we use in the FEM procedure for the purpose of map sharpening. The calculation involves two steps. Firstly, a locally averaged map (*M*_ave_) is calculated by replacing each grid point value in the original map (*M*_orig_) with the average value calculated over all 27 nearest-neighbor grid points. The sharpened map is then calculated as max(*M*_orig_ − *M*_ave_, 0). This type of sharpening has the effect of amplifying high-frequency components in the map and is useful for improving the clarity of images, although the sharpened image can also have unwanted edge effects. We note that applying unsharp masking (UM) increases map kurtosis.

**Figure 11:**
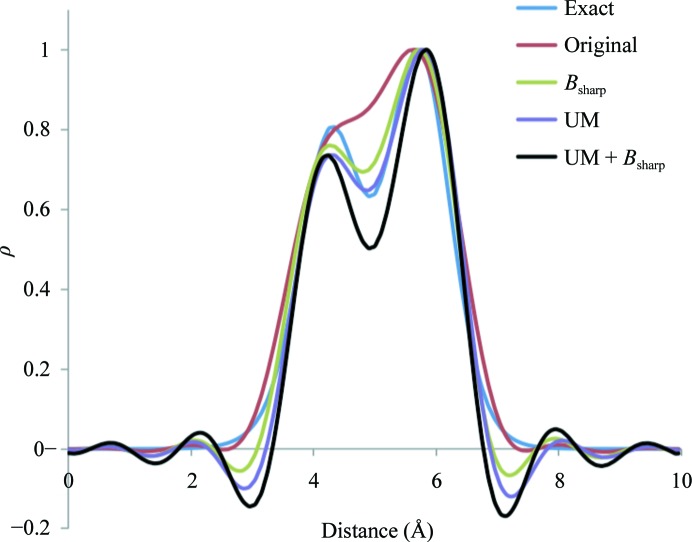
Effect of map sharpening using unsharp masking and exponential (*B*-factor) sharpening methods. See §[Sec sec2.6.2]2.6.2 for details. For easier comparison all maps are scaled to have their maximum value equal to 1.

To illustrate the effect of the two map-sharpening options, exponential and unsharp masking, we consider a test example similar to that used in the previous section: C and N atoms are placed in the middle of a *P*1 box at about a distance of 1.45 Å apart. Each atom has unit occupancy and a fixed *B* factor of 15 Å^2^. Fig. 11 ▶ shows five curves: blue is the exact electron-density distribution calculated using the Gaussian approximation formula [see, for example, formula 12 in Afonine & Urzhumtsev (2004 ▶) implemented as described in Grosse-Kunstleve *et al.* (2004 ▶)] and red is for its Fourier image calculated at 1.5 Å resolution. Both exponential (green) and unsharp masking (purple) sharpen the map similarly, while applied together they act synergistically (black).

### 2.7. Equalizing the signal: histogram equalization (quantile rank scaling)   

The goal of this step is to make the strong and weak signal in the map similar in strength. Indeed, when interpreting a map it is often less important how strong the strong signal is, as long as it is strong enough to be reliably distinguished from the noise and interpreted in terms of the atomic model. Therefore, assuming that the noise has been efficiently suppressed in a previous step, it is reasonable to expect that equalizing the map values across the unit-cell volume will yield a more easily interpretable map.

**Figure 12:**
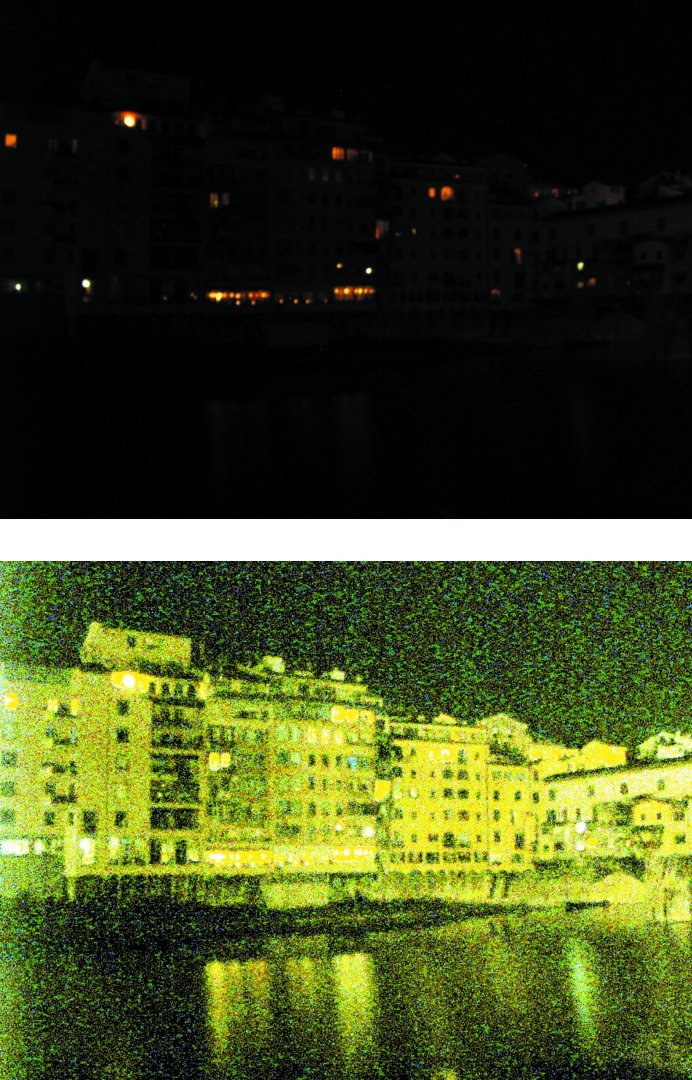
Illustration of histogram equalization. An underexposed night photograph (top) and its histogram-equalized version (bottom) (the pictures were taken by the first author). Although the HE picture is unrealistic (*i.e.* it gives a false impression that it is daytime) it does shows significantly more detail than the original image.

**Figure 13:**
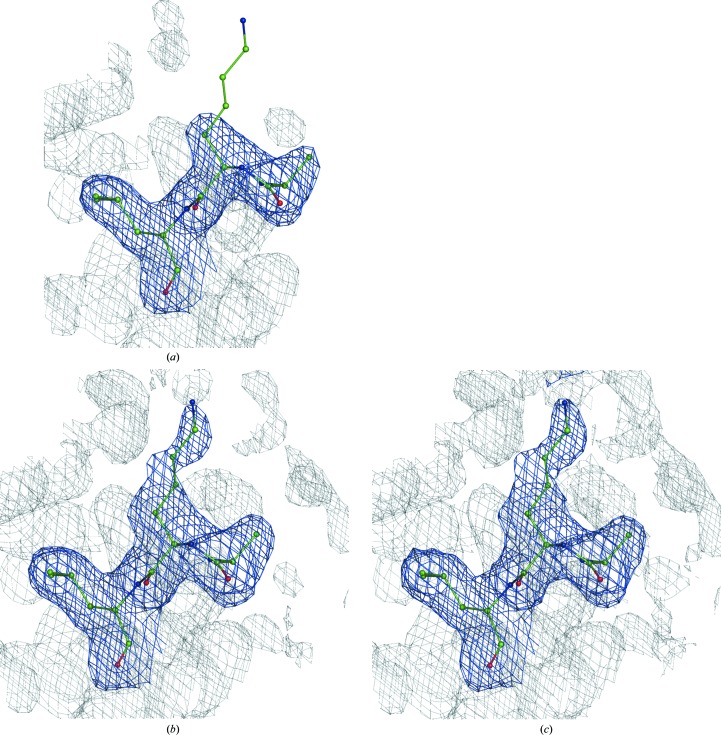
Illustration of histogram equalization. Weak side-chain density for residue Lys83 in chain *A* of PDB entry 1ssw. The synthesis in (1) was contoured at 1σ (*a*) and 0.4σ (*b*). (*c*) HE map contoured at a level equivalent to 1σ. Blue, only density within a 1.5 Å radius around atoms is shown; grey, the same as the blue map but shown within a 5 Å radius around atoms. The blue map is shown on top of the grey map.

**Figure 14:**
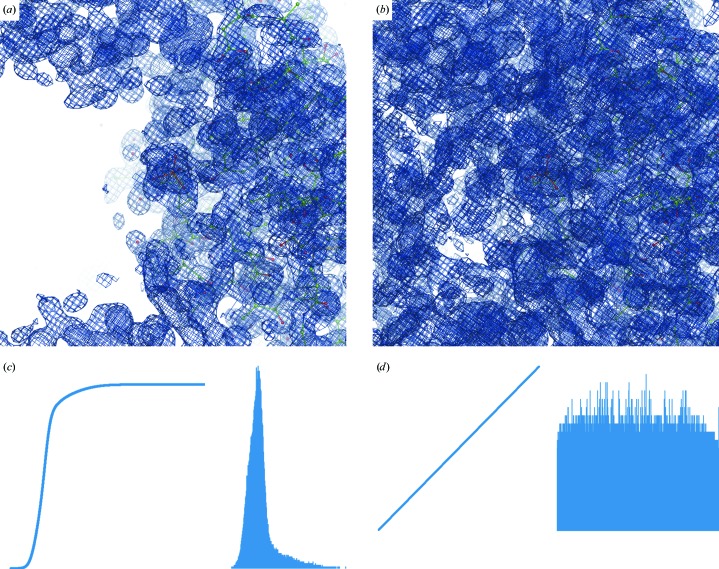
Illustration of histogram equalization for PDB entry 2bwl. First row, original map from (1) contoured at 1.3σ (left) and the HE map (right) at the same contour level. Second row, histogram and cumulative histogram of the synthesis values: left, original map; right, HE map.

**Figure 15:**
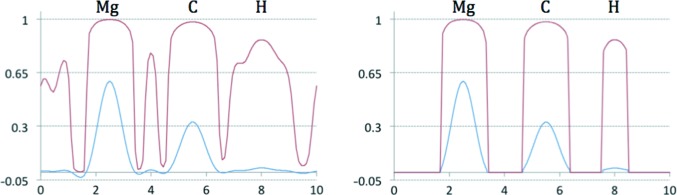
Fourier map distribution along the Mg—O—H line (blue) and its histogram-equalized version (red). Left, original map; right, map truncated with ρ_trunc_ = 0 if ρ < 0.05. Note the enhancement of noise peaks as a result of applying HE (left, red line).

The histogram-equalization (HE) method (see, for example, Hummel, 1975 ▶, 1977 ▶) is used to transform map values over the entire unit-cell volume to generate a uniform distribution of map values. This method nonlinearly (but monotonically) transforms the image values to enhance the contrast and is a routine tool in the digital image-processing field. Fig. 12 ▶ illustrates the effect of HE applied in two dimensions to a photograph. The method works similarly in three dimensions when applied to Fourier maps. After applying HE to a crystallographic map the strong and weak peaks become more equal (Fig. 13 ▶). Obviously, the noise is equalized as well (compare Figs. 14 ▶ *a* and 14 ▶ *b*), so it is essential that it is maximally removed before applying HE. Fig. 15 ▶ provides an additional (and somewhat simpler) illustration of the effect of HE on a 1 Å resolution Fourier map calculated using a test model of three atoms (Mg, O and H) placed in a *P*1 box. The three atoms were chosen specifically to yield map peaks of very different heights.

The histogram-equalization procedure consists of two steps. Firstly, the cumulative histogram of the map is calculated. Each pixel of the map is then replaced with the corresponding value taken from the cumulative histogram. This is equivalent to rescaling the map in quantile ranks as described by Urzhumtsev *et al.* (2014 ▶). The cumulative histogram of the resulting map is a straight line, which is the diagonal of a (0, 1) box (Fig. 14 ▶ *d*). Note that the HE map may be scaled in r.m.s. deviations: since this is a linear transformation, it does not modify the features of the HE map.

### 2.8. Other tools that are potentially useful in the FEM calculation procedure   

As part of developing the FEM method, we also explored other alternatives for signal equalization or enhancement and noise reduction. They are not part of the current implementation of the FEM procedure and are listed below for the sake of completeness.

We developed and tried a procedure for the combination of two maps that enhances the features that are present in both maps. This procedure is related to the minimum function that is often used in the analysis of Patterson maps (Buerger, 1970 ▶; Terwilliger *et al.*, 1987 ▶). Two maps are analyzed in this procedure, which are assumed to be on the same scale. For each threshold level ranging from *s*_1_ to *s*_2_ with a certain step, values in both maps are retained if they are simultaneously above or below the *s*_1_ + step level; otherwise, they are set to zero. The final map is the average of the two modified maps. The actual values of the thresholds *s*_1_ and *s*_2_ depend on how the maps are scaled. For example, if both maps are histogram-equalized (see §[Sec sec2.7]2.7) then *s*_1_ and *s*_2_ are equal to 0 and 1, respectively, and the step can be 0.1, for instance. The problem that we encountered with this approach is that eventually it creates ‘holes’ in the map set to zero if both values do not intersect while all surrounding grid nodes are different from zero. It may also result in the creation of sharp edges in the map, which may potentially result in strong Fourier ripples if such a map is subjected to a Fourier transform. Finally, combining maps considering two maps at a time is less powerful than the analysis of all available maps simultaneously.

We have tried other (than *B*-factor or unsharp masking) sharpening techniques such as a Kuwahara filter (a type of median filter; Kuwahara *et al.*, 1976 ▶), as has previously been reported (Diaconu *et al.*, 2005 ▶) to be useful in interpretation of low-resolution electron-microscopy maps. However, this method was not successful in our tests.

For map equalization an alternative is to partition the entire asymmetric unit into boxes, compute the r.m.s. deviation of the map in each box and then scale the map values in each box with it individually. This approach requires treating macromolecular and solvent regions separately, which is not always convenient or straightforward, and also requires knowledge of the solvent mask.

An approach that may enhance weak signal in the map and suppress noise is to apply a polynomial density modification (Hoppe & Gassmann, 1968 ▶; Collins, 1975 ▶; Raghavan & Tulinsky, 1979 ▶). Our experience was that this works well in many instances, but there are caveats. Firstly, the density map has to be truncated and rescaled so that its values are between 0 and 1, which obviously requires the application of some cutoffs, the choice of which may not always be obvious (especially automatically in software). Secondly, the transformation function has an inflection point at 0.5, which means that values below 0.5 are suppressed and values above 0.5 are enhanced. Clearly, this implies an arbitrary assumption that all peaks below 0.5 are noise and are signal otherwise.

We therefore prefer the histogram-equalization method as it efficiently equalizes the contrast while it does not require *ad hoc* parameters to be defined.

### 2.9. Note about the comparison of maps   

**Figure 16:**
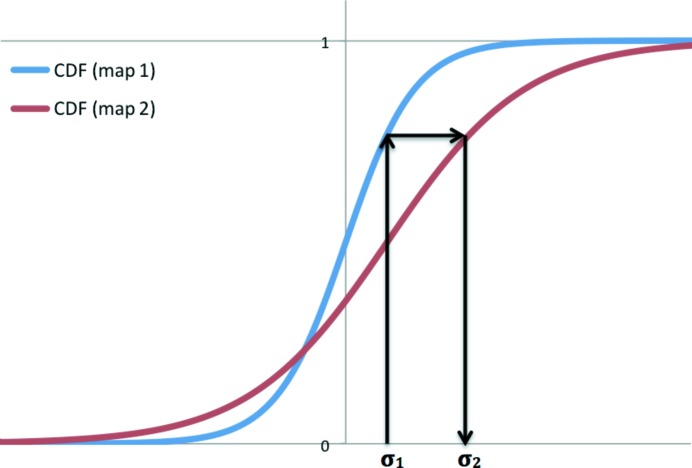
Schematic illustration of choosing equivalent map-contouring thresholds. The red and blue curves are cumulative distribution functions (CDFs) corresponding to the two r.m.s. deviation-scaled maps in question. Given the contouring threshold for map 1 (σ_1_), first one needs to find the corresponding value of the CDF. Next, one looks up the contouring threshold of map 2 (σ_2_) that corresponds to the same value of the CDF as for map 1. The contouring thresholds σ_1_ and σ_2_ encompass equivalent fractions of both maps.

In the examples below, we show maps obtained as the result of one manipulation or another. When several maps are shown it is often necessary to display them at equivalent contouring levels. Typically, this is performed by choosing a contouring threshold in r.m.s. deviations (σ) and using it for all maps that are subject to comparison. As pointed out by Urzhumtsev *et al.* (2014 ▶), this is at best suboptimal and at worst misleading, as even maps contoured at identical r.m.s. deviation thresholds may not be comparable. A better approach is to use thresholds that select equal volumes of the map, which may not correspond to identical thresholds as expressed in r.m.s. deviations (Urzhumtsev *et al.*, 2014 ▶). In the cases in the present work, if a figure shows more than one map the contouring levels are chosen such that they encompass identical volumes of the map. In practice, obtaining equivalent contouring levels expressed in r.m.s. deviations is achieved in the following way. Firstly, two cumulative distribution functions (CDFs) are calculated from the two maps. Then, given the selected contouring level for the first map, the corresponding CDF value is obtained and this value is used to obtain the corresponding argument for the CDF of the second map, which is the desired threshold value (see Fig. 16 ▶). This can be performed using the *phenix.map_comparison* tool, which implements the methodologies described in Urzhumtsev *et al.* (2014 ▶).

## 3. Results   

Unlike density modification (classical or statistical), the FEM is not an iterative phase-improvement procedure and it is generally not expected to reveal features that are not already present in the original map from (1). Instead, it makes the available signal more apparent by suppressing the noise and by equalizing the magnitude of the weak signal with the strong signal.

The FEM procedure can be applied to X-ray and nuclear density maps and has been tested at resolutions from high (1 Å) to low (about 4 Å). The FEM protocol was designed and optimized for maps corresponding to near-to-final models when the noise is usually smaller than the signal. For initial and intermediate cases the amount and magnitude of the noise may be comparable with the signal, making the noise very difficult to suppress efficiently. In such cases FEM maps should be used with care. However, FEM maps are expected to be less model-biased than the starting maps as they are filtered by the composite residual OMIT map.

Keeping Fig. 15 ▶ in mind, we note that FEM maps remain similar over a broad range of contour levels. If the FEM is scaled by the r.m.s. deviation [which is likely to be the default in many molecular-graphics tools such as *Coot* (Emsley *et al.*, 2010 ▶) and *MAIN* (Turk, 2013 ▶)], then thresholds between 0 and 1σ are generally desirable. Also, it is important to note that synthesis (1) and the FEM are likely to be less comparable at identical contouring levels (§[Sec sec2.9]2.9).

Below, we provide a number of examples of applying the FEM to selected structures that illustrate typical outcomes of the procedure. In the majority of cases the FEM map shows previously missing density for side chains, enhanced density for disordered loops and alternative conformations. FEM maps are typically cleaner overall and flatter in bulk-solvent regions. They are also a better target for real-space refinement.

### 3.1. PDB entry 1f8t: better side-chain density   

**Figure 17:**
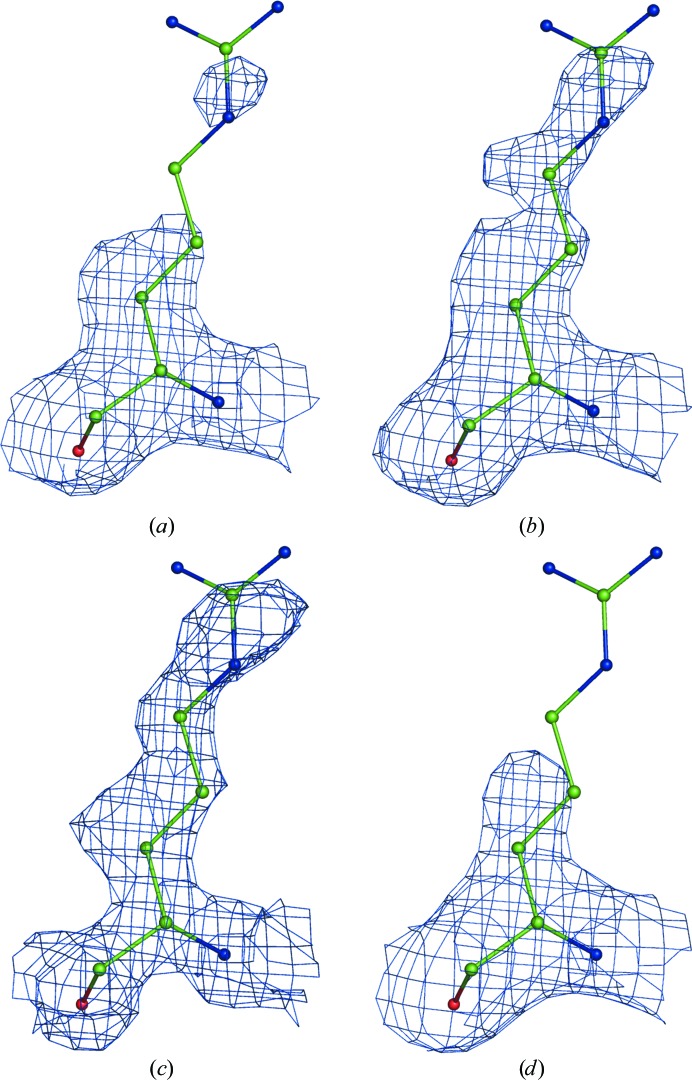
Maps for PDB entry 1f8t residue Arg74 (chain *L*). (*a*) Map from (1) at 1.0σ. (*b*) Composite residual OMIT map from (2) at 1.5σ calculated as described in §[Sec sec2.4]2.4. (*c*) FEM contoured at 1.1σ. (d) *RESOLVE* density-modified map at 0.8σ.

Fig. 17 ▶ shows no density (above the 1σ threshold) for the side chain of Arg74 (chain *L*) in the map from (1). At the same time, the composite residual OMIT map calculated with (2) as described in §[Sec sec2.4]2.4 shows side-chain density at a level of 1.5σ. FEM reveals density for the entire residue at a level equivalent to 1σ. For comparison, we calculated a *RESOLVE* density-modified map, which did not show any density above the 0.8σ level.

### 3.2. PDB entry 1nh2: cleaning an overall noisy map   

**Figure 18:**
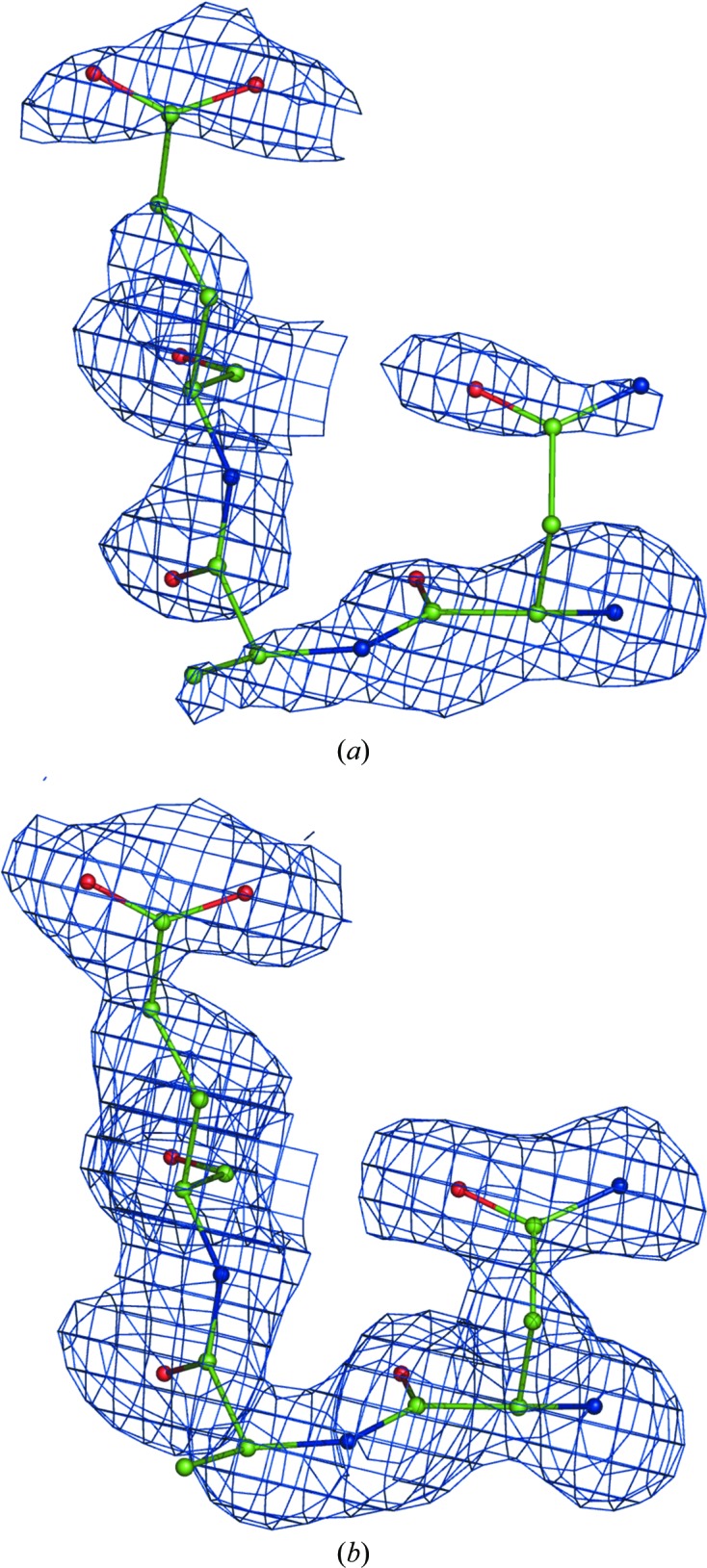
Maps for PDB entry 1nh2. (*a*) Map from (1) at 1.0σ. (*b*) FEM contoured at the equivalent to 1.0σ. Residues 3–5 of chain *B* are shown.

**Figure 19:**
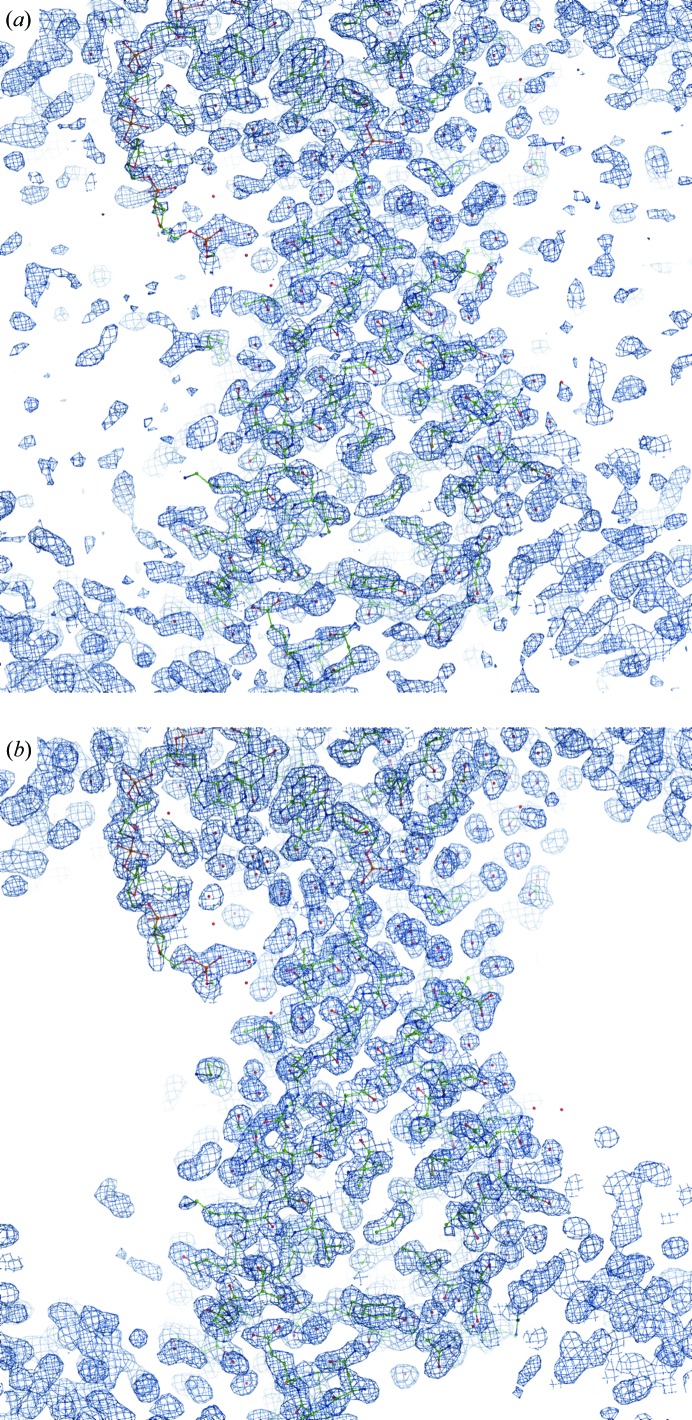
View of macromolecule and bulk-solvent areas for PDB entry 1nh2. The map from (1) (*a*) and the FEM (*b*) contoured at 1.0σ. The macromolecule and the bulk-solvent interface are shown.

The map from (1) is unusually noisy and notoriously patchy for this structure. Fig. 18 ▶ shows an example of typical map improvement throughout the entire model. Fig. 19 ▶ provides an overall view of the macromolecule–solvent interface and shows significant noise reduction in the bulk-solvent region as a result of applying FEM.

### 3.3. PDB entry 2r24: the structure of aldose reductase obtained using neutron data   

**Figure 20:**
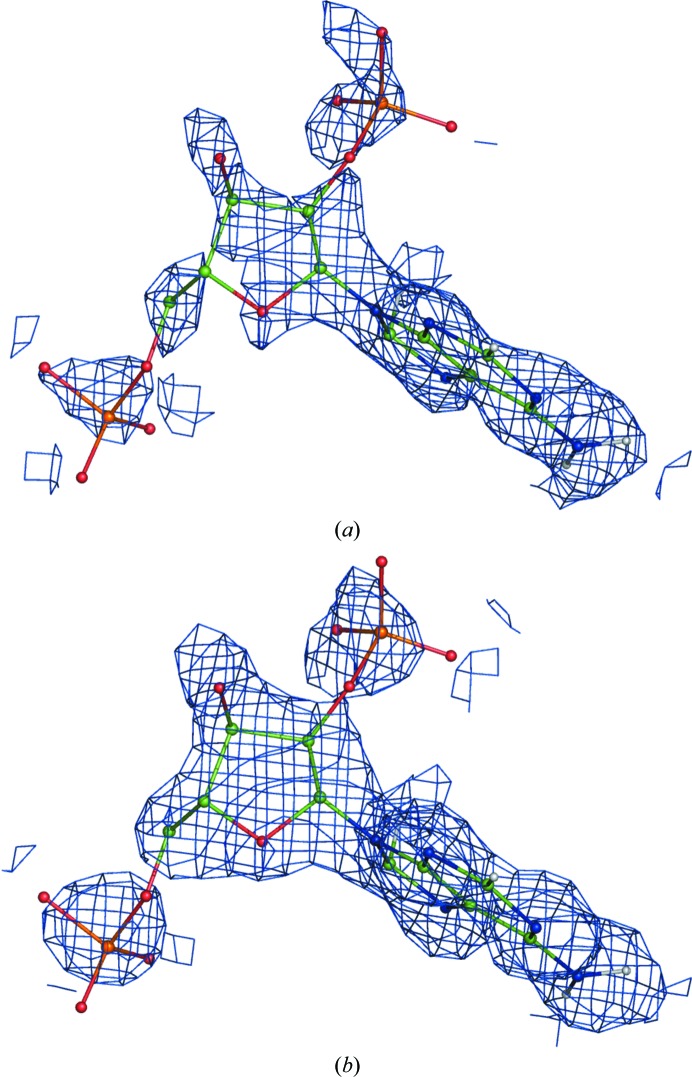
Neutron structure of aldose reductase (PDB entry 2r24). A fragment of the NAP ligand in the nuclear map is shown. (*a*) is the map from (1) and (*b*) is the FEM; both are contoured at 1σ.

We have examined the outcomes of FEM calculations for about a dozen neutron structures selected from the PDB. The general observations stated for X-ray structures also hold for structures refined against neutron data. Fig. 20 ▶ illustrates the application of FEM to a neutron structure. In this example the somewhat broken ligand density map from (1) improves after calculating the FEM.

### 3.4. PDB entry 2g38: a highly anisotropic data set   

**Figure 21:**
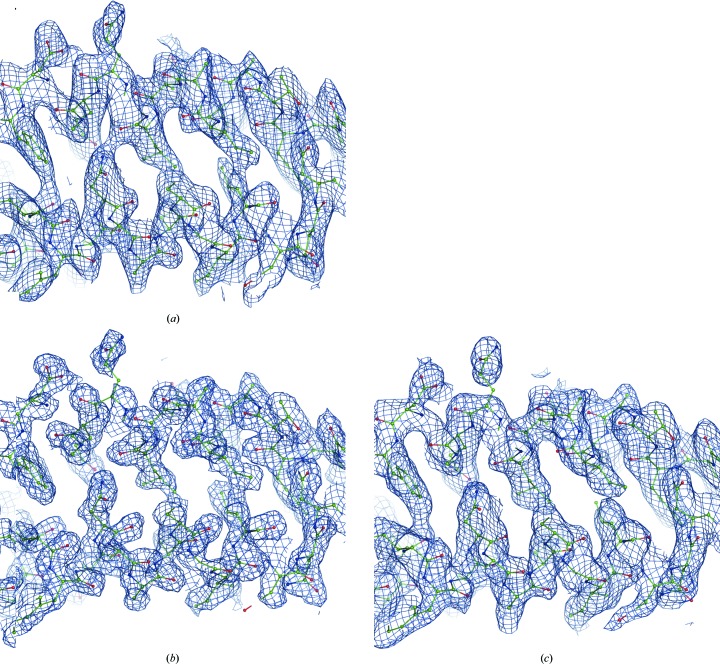
(*a*) The original map from (1), (*b*) the FEM and (*c*) a *RESOLVE* density-modified map for PDB entry 2g38, where the data are known to be affected by severe anisotropy. Note the reduction in the effect of map anisotropy along the vertical direction in both the FEM and *RESOLVE* maps.

This is a classical example of data with severe anisotropy and has served as an example for the *Anisotropy Correction Server* (Strong *et al.*, 2006 ▶). The original diffraction data were kindly provided by the author (Michael Sawaya), as the corresponding PDB entry contains only an anisotropy-corrected version of the measured intensities. Fig. 21 ▶ shows three maps: the original map from (1), the FEM and a density-modified *RESOLVE* map. The latter two maps show a notable reduction in anisotropy effects (elongation of map contours along the vertical direction).

### 3.5. PDB entry 1se6: an incorrectly placed ligand   

**Figure 22:**
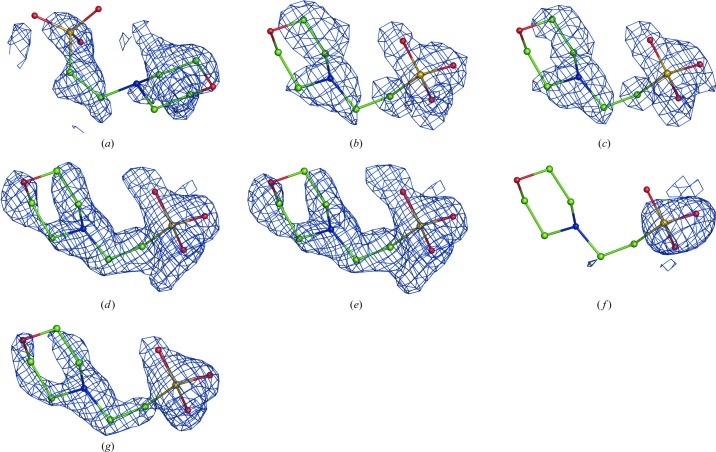
An example of a misfitted ligand in PDB entry 1se6. (*a*) The incorrect fit shown in the map from (1). The FEM is shown calculated with (*b*) and without (*c*) the incorrectly fitted ligand included in the calculations. The composite residual OMIT map is shown with (*d*) and without (*e*) the incorrectly fitted ligand included in the calculations. (*f*) *RESOLVE* density-modified map. (*g*) Ligand-omit map from (1) after correcting the ligand fit followed by a round of refinement. All maps are shown at 1σ or equivalent.

Pozharski *et al.* (2013 ▶) pointed out a misfitted ligand in this entry. While the Fourier map from (1) does not clearly identify the ligand at 1σ (Fig. 22 ▶ *a*), feature-enhanced maps calculated both with (Fig. 22 ▶ *b*) and without (Fig. 22 ▶ *c*) the ligand present in the input model file better identified the ligand. Also, composite residual OMIT maps calculated with (Fig. 22 ▶ *d*) and without (Fig. 22 ▶ *e*) the ligand both unambiguously confirm its identity. A *RESOLVE* density-modified map barely shows the ligand density. Correcting the ligand fit is obvious based on the residual OMIT or FEM maps, and a subsequent round of refinement yields a clear ligand-omit Fourier map from (1).

### 3.6. PDB entries 2vui and 2ppi: the importance of map sharpening   

**Figure 23:**
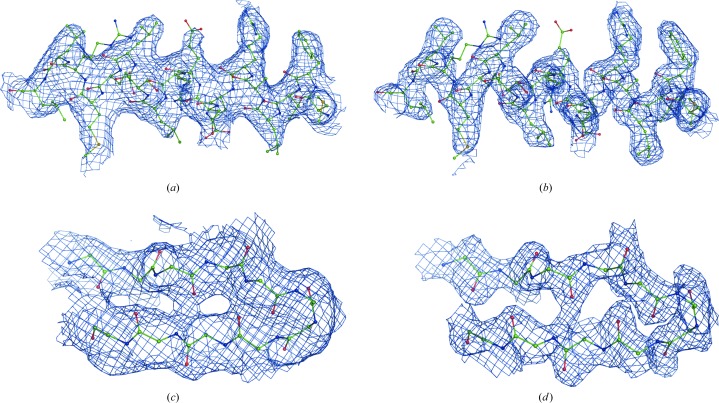
Feature-enhanced maps (FEMs) without sharpening (*a*, *c*) and with sharpening (*b*, *d*). (*a*, *b*) PDB model 2vui, residues 123–140 of chain *B*. (*c*, *d*) PDB model 2ppi, residues 32–43 of chain *A*. All maps are contoured at 1σ.

The two examples in Fig. 23 ▶ illustrate the importance of using map sharpening. Typically, sharpening deblurs the image, making the features more distinctive, and often reveals features that are not otherwise visible, such as residue side chains. As expected, sharpening does increase the map noise; however, when applied as part of the FEM protocol the noise arising from sharpening is mostly eliminated.

### 3.7. PDB entry 3i9q: FEM as a better target for real-space refinement   

**Figure 24:**
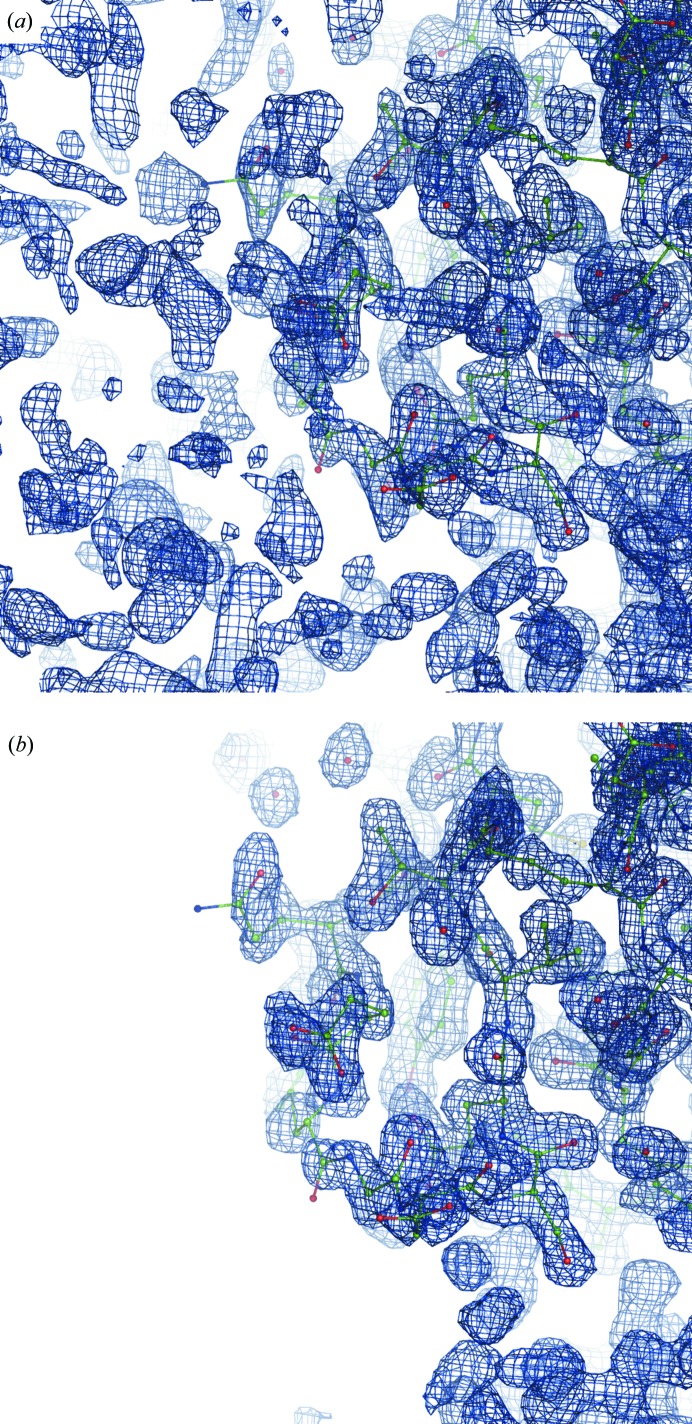
(*a*) Map from (1) and (*b*) FEM for PDB entry 3i9q, both contoured at 1σ.

**Figure 25:**
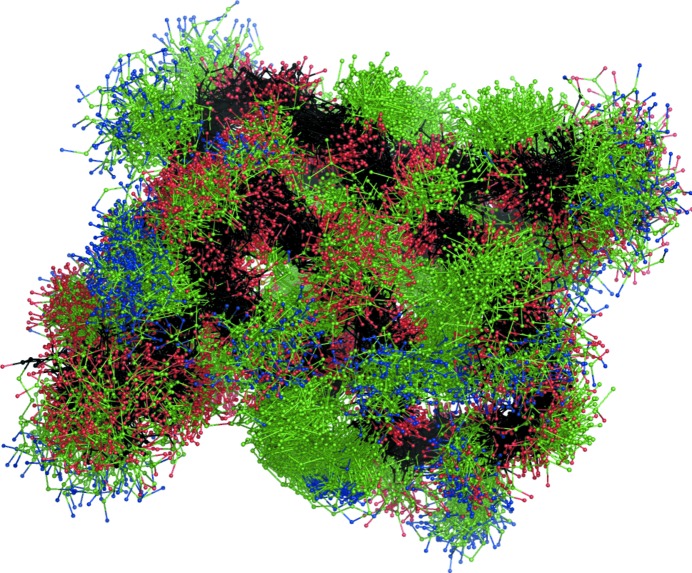
The first 100 models from an ensemble of 1000 MD-generated models, illustrating the diversity of starting points for real-space refinement (see §[Sec sec3.7]3.7 for details). Backbone atoms are shown in black.

**Figure 26:**
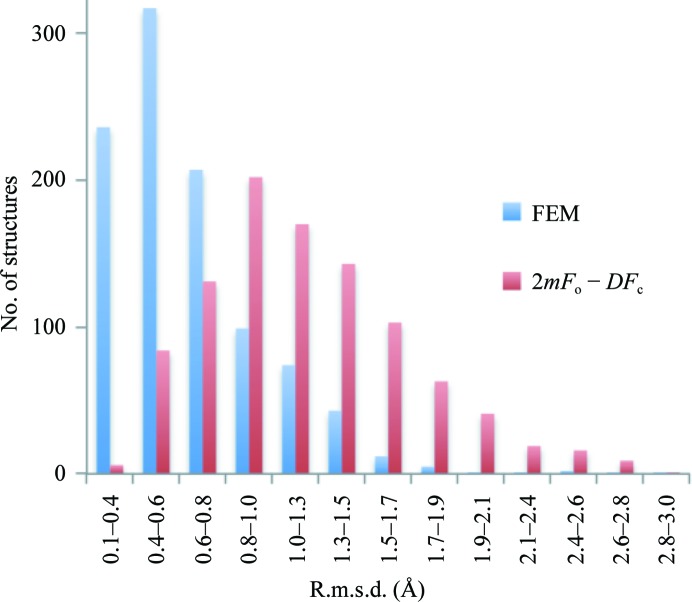
Distribution of r.m.s. deviations between real-space refined models and the best available model: refinements against FEM (blue) and against the map from (1) (red). Clearly, the majority of structures refined closer to the true structure when the FEM was used as a target map.

To further illustrate the FEM procedure, we performed the following test. We selected PDB entry 3i9q (data resolution 1.45 Å), which we re-refined using *phenix.refine* (Afonine *et al.*, 2012 ▶). Using this model, we calculated two maps: the FEM and the usual map from (1) (Figs. 24 ▶ *a* and 24 ▶ *b*). Since we wanted to compare the original σ_A_ map (Read, 1986 ▶) with the FEM, no modeling of missing reflections, anisotropy correction or sharpening was performed. What is remarkable about this structure is that the map from (1) is outstandingly poor, perhaps owing to poor completeness in the low-resolution zone (50% completeness in the 17.49–6.58 Å zone). We then removed everything but the macromolecule from the model and subjected this model to 1000 independent molecular-dynamics (MD) simulation runs using *phenix.dynamics*, with each run continuing until the root-mean-square deviation (r.m.s.d.) between the starting and current models exceeded 3 Å. This generated a diverse ensemble of 1000 structures as shown in Fig. 25 ▶. Next, each model from the ensemble was subjected to ten macrocycles of real-space refinement using *phenix.real_space_refine* (Afonine, Headd *et al.*, 2013 ▶) against the FEM and the map from (1) independently. Each refinement macrocycle included model morphing (Terwilliger *et al.*, 2012 ▶), simulated annealing with a slow-cooling protocol starting from 5000 K and local and overall gradient-driven minimization (Afonine *et al.*, 2012 ▶). The refinement success was quantified by calculation of the r.m.s. deviation between the unperturbed model (before MD) that we consider to be the best available model and the model after real-space refinement. Fig. 26 ▶ shows a histogram of r.m.s. deviations for the refinement outcomes against the FEM and the map from (1). Clearly, in a majority of cases refinement against a feature-enhanced map resulted in refined models that were significantly closer to the true structure than those refined against a standard map.

## 4. Conclusions   

The Fourier maps routinely used in crystallographic structure solution are never perfect owing to errors in both the experimental data and the parameters of the structural model. Various kinds of map errors may impede structure solution and completion or result in erroneous map interpretation, which in turn leads to an incorrect atomic model.

Over the decades, great effort has been put into the development of methods to improve crystallographic maps. However, most existing methods only address one or a few map quality-related problems at a time. Also, some of the existing methods designed to address one problem may make other problems worse. More thorough and efficient methods are typically very computationally expensive (they may take hours or days to compute) and also may require case-specific parameterization as they use refinement or model building.

In this manuscript, we have presented a novel method of crystallographic map modification that simultaneously combines several desirable maps, requires minimal inputs (*i.e.* the current atomic model and diffraction data), does not require time-consuming calculations (such as refinement or model building) and is relatively fast to calculate (from less than a minute to a few minutes). We call the map obtained as a result a feature-enhanced map (FEM).

All of the key tools used in the FEM calculation (including the FEM protocol itself) are implemented as part of *cctbx*. The calculation of OMIT maps is available as a command-line tool called *phenix.composite_omit_map* (Echols & Afonine, 2014 ▶). The FEM calculation shown here is available in *PHENIX* (Adams *et al.*, 2010 ▶) starting with version dev-1832 from the command line (phenix.fem) and in the *PHENIX* GUI. The data and scripts used to obtain the figures in this manuscript are available at http://phenix-online.org/phenix_data/.
